# Identification and analysis of type 2 diabetes-mellitus-associated autophagy-related genes

**DOI:** 10.3389/fendo.2023.1164112

**Published:** 2023-05-08

**Authors:** Kun Cui, Zhizheng Li

**Affiliations:** ^1^ Respiratory Medicine, Tangshan Gongren Hospital, Tangshan, Hebei, China; ^2^ Department of Respiratory and Critical Care Medicine, Tangshan Gongren Hospital, Tangshan, Hebei, China

**Keywords:** autophagy, differentially expressed genes, hub genes, type 2 diabetes mellitus, biomarker

## Abstract

**Introduction:**

Autophagy, an innate safeguard mechanism for protecting the organism against harmful agents, is implicated in the survival of pancreatic â cells and the development of type 2 diabetes mellitus (T2DM). Potential autophagy-related genes (ARGs) may serve as potential biomarkers for T2DM treatment.

**Methods:**

The GSE25724 dataset was downloaded from the Gene Expression Omnibus (GEO) database, and ARGs were obtained from the Human Autophagy Database. The differentially expressed autophagy-related genes (DEARGs) were screened at the intersection of ARGs and differentially expressed genes (DEGs) between T2DM and non-diabetic islet samples, which were subjected to functional enrichment analyses. A protein–protein interaction (PPI) network was constructed to identify hub DEARGs. Expressions of top 10 DEARGs were validated in human pancreatic â-cell line NES2Y and rat pancreatic INS-1 cells using quantitative reverse transcription polymerase chain reaction (qRT-PCR). Cell viability and insulin secretion were measured after cell transfection with lentiviral vector EIF2AK3 or RB1CC1 into islet cells.

**Results:**

In total, we discovered 1,270 DEGs (266 upregulated and 1,004 downregulated genes) and 30 DEARGs enriched in autophagy- and mitophagy-related pathways. In addition, we identified GAPDH, ITPR1, EIF2AK3, FOXO3, HSPA5, RB1CC1, LAMP2, GABARAPL2, RAB7A, and WIPI1 genes as the hub ARGs. Next, qRT-PCR analysis revealed that expressions of hub DEARGs were consistent with findings from bioinformatics analysis. EIF2AK3, GABARAPL2, HSPA5, LAMP2, and RB1CC1 were both differentially expressed in the two cell types. Overexpression of EIF2AK3 or RB1CC1 promoted cell viability of islet cells and increased the insulin secretion.

**Discussion:**

This study provides potential biomarkers as therapeutic targets for T2DM.

## Introduction

Diabetes mellitus, a multifactorial disease that involves environmental and genetic factors, is primarily divided into type 1 and type 2 diabetes mellitus (T2DM) (1). More than 90% of diabetes is T2DM, which is a chronic metabolic disease, manifested by high glucose levels induced by insulin resistance, defects in insulin secretion, or inadequate compensatory insulin secretory response (2, 3). It has been widely accepted that various processes including apoptosis, oxidative stress, inflammation, and endoplasmic reticulum stress were closely associated with T2DM (4–7). T2DM may increase the risk of mortality or other cardiovascular diseases (3). At present, the incidence of T2DM has been increasing yearly and shows a younger trend, which has become a worldwide public health problem (8, 9). These challenges necessitate the discovery of promising therapeutic targets for T2DM.

Autophagy is a catabolic process, which is ubiquitous in eukaryotes (10). It can be stimulated by various stresses and quickly induces a response to clear impaired organelles and protein aggregates *via* autophagosomes, which are then delivered to and degraded in lysosomes (11, 12). Defects in autophagy are involved in the development of several human diseases, such as cancer, cardiovascular diseases, neurodegenerative diseases, and diabetes mellitus (13, 14). It has been recognized that autophagy is crucial in T2DM development, and it protects the structure and function of pancreatic β cells, therefore leading to the survival of pancreatic β cells (14). The autophagy process is mediated by autophagy-related genes (ARGs). For instance, ATG5 and LC3B are found to be decreased in diabetes mellitus patients with or without complications, indicating that downregulated ATG5 and LC3B may be implicated in the deficiencies of autophagy (15). A recent study has deciphered that upregulated ARGs such as Atg16L1, Atg16L2, and GabarapL1 are closely associated with the comorbidity of Alzheimer’s disease and T2DM (16). Therefore, further investigation of ARGs in T2DM may be helpful in discovering novel biomarkers as preventive or therapeutic targets for T2DM.

In the past few decades, integrated analysis based on transcriptome data derived from some databases have become a useful approach to identify novel differential expressed genes (DEGs) and reveal their biological functions in diseases (17). In this study, we screened the differentially expressed ARGs (DEARGs) at the intersection of ARGs and differentially expressed genes (DEGs) between T2DM and non-diabetic islet samples. Correlation analysis and functional enrichment analysis were performed for the DEARGs. A protein–protein interaction (PPI) network was generated to identify hub DEARGs. Additionally, expressions of key DEARGs were measured, and *in vitro* experiments were carried out to validate the role of key DEARGs in T2DM.

## Materials and methods

### Microarray data and autophagy-related genes datasets

Microarray expression profiles of GSE25724 dataset were downloaded from the Gene Expression Omnibus (GEO, https://www.ncbi.nlm.nih.gov/geo/) database on the GPL96 platform (HG-U133A, Affymetrix Human Genome U133A Array), including seven non-diabetic islet samples and six T2DM islet samples. A total of 222 ARGs were acquired from the Human Autophagy Database (http://autophagy.lu/clustering/index.html).

### Screening of DEARGs

Before analysis, principal components analysis (PCA) was used to evaluate the distribution of normal islet samples and T2DM islet samples. In order to improve the data quality, the low-quality samples were removed based on the PCA results. In addition, we evaluated the standardization of gene expression in GSE25724 dataset to determine whether the gene expression of each islet sample was relatively consistent. Differential expression analysis was performed between normal samples and T2DM islet samples by using the Bioconductor’s “Limma” package (18). Candidate genes with *p* < 0.05 and |log fold change (FC)| ≥1 were considered as DEGs. Furthermore, DEGs with logFC > 0 were upregulated, while those with logFC < 0 were considered as downregulated. The volcano plots and heatmap were plotted using an online bioinformatics analysis platform (https://www.bioinformatics.com.cn). A Venn diagram was generated using the online Venn diagrams tool (Venny 2.1, https://bioinfogp.cnb.csic.es/tools/venny/) to identify DEARGs at the intersection of DEGs and ARGs.

### Functional enrichment analysis of DEARGs

“ClusterProfiler” (19) in R (https://bioconductor.org/packages/clusterProfiler/) was used for Kyoto Encyclopedia of Genes and Genomes (KEGG) pathway and Gene Ontology (GO) functional enrichment analyses. KEGG pathway and module were directly obtained using KEGG API. GO annotation data were obtained directly from Bioconductor OrgDb packages. The catalogues of GO terms enrichment analysis included biological process (BP), cellular component (CC), and molecular function (MF). The threshold of *p*-value < 0.05 was regarded as statistically significant. The results of enrichment analysis were visualized using “Enrichment map” R package (20).

### PPI network construction of DEARGs

The STRING database, an online tool, was used to construct a PPI network of DEARGs. A confidence score >0.4 was used as the cutoff to screen the PPI pairs. The PPI network was visualized using Cytoscape (Version 3.7.2). To identify the tightly connected protein clusters, molecular complex detection (MCODE) algorithm in Cytoscape was employed, with degree cutoff=2, node score cutoff=0.2, k-core=2, and max.depth=100. Nodes within this complex network were ranked based upon their degree centrality values using the CytoHubba plugin. Hub DEARGs were further determined based on the top 10 connectivity degree values.

### Analysis of 10 key hub DEARGs

To investigate the differences in expressions of key hub DEARGs, we evaluated their gene expression between normal and T2DM and visualized using boxplots generated from ImageGP (http://www.ehbio.com/ImageGP/). The correlation between these hub DEARGs were analyzed using Spearman correlation analysis. The *p*-value < 0.05 was considered as statistically significant.

### Cell culture

The human pancreatic β-cell line NES2Y and rat pancreatic INS-1 cells were used. Cells were inoculated in six-well plates (1 × 10^5^ cells/well) containing 1640 medium with 10% fetal bovine serum. Incubation was done for 12 h at 37°C in a 5% CO_2_ atmosphere. Cells were starved for 16 h followed by streptozotocin (STZ, 5 mM) treatment for a further 24 h and then were collected.

### Quantitative reverse transcription polymerase chain reaction

Meanwhile, we performed qRT-PCR to quantify the expression of 10 hub DEARGs in NES2Y and INS-1 cells with or without STZ treatment. RNA extraction from cells was performed using TRIzol reagent (Invitrogen, 15596-026) and then reverse-transcribed into cDNA using Hiscript II QRT Supermix for qPCR kits (Vazyme, China) following the instructions. A ChamQ Universal SYBR kit (Vazyme, China) was implemented for qPCR on the Bio-Rad CFX96 Real-Time PCR Detection System with the thermocycler conditions: 95°C for 2 min, followed by 35 cycles of 95°C for 10 s and 60°C for 30 s. Beta-actin was used as the internal reference gene (21). Relative mRNA expression was calculated using the 2^−△△Ct^ method. △△Ct = (Ct, target gene − Ct, reference gene) − (Ct, target gene − Ct, reference gene) control. Experiments were conducted in triplicate. Primer3plus (https://www.primer3plus.com) was used to design all primer sequences (**Table 1**).

**Table 1:** Primer sequences used in qRT-PCR.

Genes	Forward (5’-3’)	Reverse (5’-3’)
EIF2AK3	CAGCTGTGCAGGAAGGAGAA	CGACTTGTCCCGTGTGTGTA
WIPI1	CTTCAAGCTGGAACAGGTCACC	CGGAGAAGTTCAAGCGTGCAGT
FOXO3	CCAGTGACTTGGACCTGGAC	TCCCCACGTTCAAACCAACA
GABARAPL2	TGGAACACAGATGCGTGGAA	GTTAGGCTGGACTGTGGGAC
GAPDH	GTCTCCTCTGACTTCAACAGCG	ACCACCCTGTTGCTGTAGCCAA
HSPA5	GTGCCCACCAAGAAGTCTCA	ATTTCTTCAGGGGTCAGGCG
ITPR1	GGTAGAGACGGGGGAGAACT	GAGCACATCTCCTACTCCGC
LAMP2	GTGCAACAAAGAGCAGGTGG	TGATGGCGCTTGAGACCAAT
RAB7A	AGTTTCTCATCCAGGCCAGC	CTTGGCACTGGTCTCGAAGT
RB1CC1	CCGTCTCCCATCCAAACACA	GTCCTGCATGCGCCTACTAT
β-actin	CCACCATGTACCCAGGCATT	CGGACTCATCGTACTCCTGC

### Lentiviral vector construction and transfection

The lentivirus vector for overexpressing EIF2AK3 (Lv-EIF2AK3), RB1CC1 (Lv-RB1CC1), and their corresponding negative control lentivirus (Lv-NC) were constructed from Hanbio (Shanghai, China). NES2Y cells or INS-1 cells were seeded into a 24-well plate at 70%–80% confluence and were transfected with Lv-EIF2AK3, Lv-RB1CC1, or Lv-NC at a multiplicity of infection (MOI) of 5 in the presence of 10 μg/ml polybrene. The procedure of transfection was performed using Lipofectamine 2000 (Invitrogen, USA) according to the manufacturer’s instructions. Transfection efficiency was verified by Western blotting after 72 h of transfection.

### Western blot

The total protein was extracted from the NES2Y and INS-1 cells and quantitated with a BCA Kit (Beyotime, China). Proteins was separated on 10% sodium dodecyl sulfate–polyacrylamide gel electrophoresis (SDS-PAGE) and transferred onto polyvinylidene fluoride (PVDF) membrane. Then, 5% skimmed milk in Tris-buffered saline with Tween 20 (TBST) buffer was used to block the membranes and inoculated overnight with the following primary antibodies: EIF2AK3 (20582-1-AP; Proteintech), GABARAPL2 (DF10158; Affinity), HSPA5 (AF5366; Affinity), LAMP2 (66301-1-Ig; Affinity), RB1CC1 (17250-1-AP; Affinity), and β-actin (AF7018; Affinity) at 4 °C. After washing three times with 0.1% TBST, we then used horseradish peroxidase (HRP)-labeled secondary antibody (S0001; Affinity) to probe the membranes for 1 h at room temperature. Subsequently, the signals were quantified with the assistance of ImageJ software. β-Actin was used as an internal control.

### Cell viability assay

The propidium iodide (PI) staining and Cell Count Kit-8 (CCK-8) assay was performed to measure the cell viability. After transfection, the cells were seeded in 96-well plates at 1 × 10^4^ cells per well and culture at 37°C with 5% CO_2_ for 24 h. Then, the STZ was added and incubated for another 24 h for cell viability analysis. For PI staining, the cells were collected and washed with phosphate-buffered saline (PBS), then treated with PI staining solution (50 μg/ml) for 15 min. Subsequently, the cells were counterstained with DAPI solution and viewed using a fluorescence microscope (Olympus) to capture images. For CCK8, a total of 10 µl CCK-8 solution (Dojindo Laboratories) was added to each well. Following incubation for 2 h at room temperature, the absorbance was measured at 450 nm with a microplate reader (Bio‐Tek).

### Insulin secretion

The cells were transfected with lentivirus and then cultured in 96-well plates, followed by incubation at 37°C with 5% CO_2_ for 24 h. Next, cells were treated with STZ for 24 h. Concentrations of insulin released from culture cells were determined by enzyme-linked immunosorbent assay (ELISA, Millipore) according to the manufacturers’ instructions.

### Statistics analysis

Experimental data were analyzed by using GraphPad Prism 8.0 software (GraphPad Prism Software Inc., CA, USA). Data were expressed means ± standard deviation. Statistical analysis was conducted using unpaired Student’s t-test and one-way analysis of variance (ANOVA). The value of *p* < 0.05 was considered as statistically significant.

## Results

### Screening DEGs between normal and T2DM islet samples

**Figure 1:**
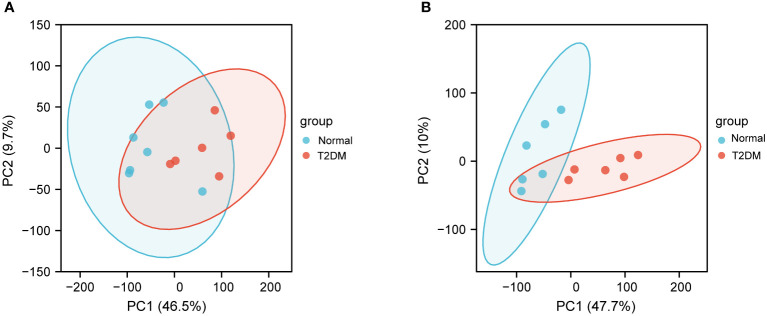
Principal components analysis (PCA) of GSE25724. **(A)** PCA of normal and T2DM islet samples before removing the outliers. **(B)** PCA after removing the outliers. The PCA1 represents the first principal component, and the PCA2 represents the second principal component.

**Figure 2:**
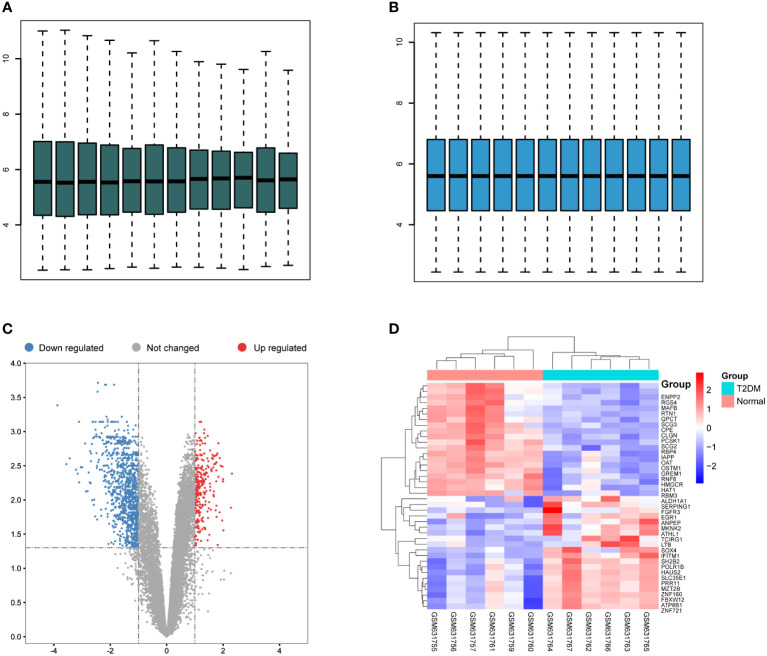
Differentially expressed gene (DEGs) in T2DM and normal islet samples. **(A)** Box plots of the gene expression data before normalization. **(B)** Box plots for gene expression data after normalization. **(C)** Volcano plots for DEGs between T2DM and normal islet samples. **(D)** Heatmap of the expression patterns of the DEGs.

To investigate the underlying mechanisms of the onset of T2DM, we downloaded RNA-seq data of six T2DM and seven normal islet samples from GEO database (GSE25724). We first conducted principal components analysis (PCA) analysis to determine whether there was a distinct expression pattern between T2DM and normal groups. As shown in **Figure 1**, one sample was not well clustered. We deleted the low-quality sample (GSM631757) and normalized the expression levels of the remained samples to better performed further analyses (**Figures 2A, B**). A total of 1,270 DEGs including 266 upregulated DEGs and 1,004 downregulated genes (|logFC|>1 and adjust *p* < 0.05, **Figure 2C**). Next, we performed heatmap analysis with top 20 upregulated genes and 20 downregulated genes. We found that there was indeed a different expression pattern between T2DM and normal groups (**Figure 2D**).

### Identification of DEARGs in T2DM

**Figure 3:**
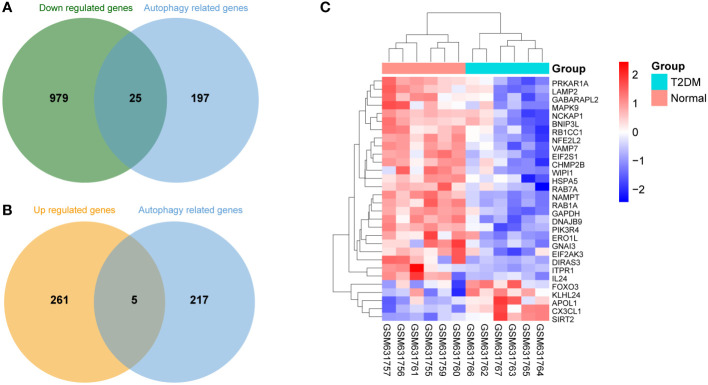
Differentially expressed autophagy-related genes (DEARGs) in T2DM and normal islet samples. **(A)** Venn analysis of 25 downregulated DEARGs. **(B)** Venn analysis of five upregulated DEARGs. **(C)** Heatmap of the expression patterns of the 30 DEARGs.

Based on the aforementioned DEGs and 222 ARGs, 30 DEARGs were identified at the intersection between DEGs and ARGs (**Table 2**), including 25 downregulated genes (**Figure 3A**) and 5 upregulated genes (**Figure 3B**). Their corresponding biological functions were detailed in **Supplementary Table S1**. Furthermore, a heatmap was used to visualize the relative expression patterns of 30 DEARGs between T2DM and normal samples. Most of DEARGs were downregulated in T2DM samples, and five DEARGs such as FOXO3, KLHL24, APOL1, CX3CL1, and SIRT2 were increased in T2DM samples (**Figure 3C**).

**Table 2:** The 30 differentially expressed autophagy-related genes.

DEARGs	logFC	AveExpression	p.Value	adj.p.Value	Changes
KLHL24	1.544727	9.076108	0.000494	0.006425	Up
SIRT2	1.379236	8.254223	0.000283	0.004947	Up
CX3CL1	1.325203	7.00778	0.000211	0.004262	Up
APOL1	1.24108	6.740273	0.004194	0.020094	Up
FOXO3	1.153503	9.341613	8.28E-06	0.001233	Up
ITPR1	-1.06092	3.611072	0.001725	0.012334	Down
RAB7A	-1.07815	6.721588	0.010801	0.036076	Down
ERO1L	-1.0937	5.559489	0.003534	0.018238	Down
GABARAPL2	-1.11002	8.036322	0.005566	0.023972	Down
EIF2AK3	-1.12623	5.294139	0.002972	0.016517	Down
NCKAP1	-1.17705	7.720637	0.016877	0.048382	Down
RB1CC1	-1.21342	6.388162	0.003535	0.018238	Down
GNAI3	-1.21642	6.367202	0.002967	0.016511	Down
GAPDH	-1.24245	11.31524	0.000103	0.003165	Down
EIF2S1	-1.26452	7.342713	0.000849	0.008367	Down
MAPK9	-1.28257	6.075861	0.00391	0.01929	Down
IL24	-1.30371	6.414092	0.017592	0.049795	Down
DIRAS3	-1.33308	4.837824	0.000722	0.007739	Down
NFE2L2	-1.36507	7.639979	0.000639	0.007337	Down
LAMP2	-1.39244	6.273228	0.002958	0.01649	Down
HSPA5	-1.43291	8.907091	0.016117	0.04689	Down
DNAJB9	-1.54165	7.998084	0.00042	0.005924	Down
WIPI1	-1.54636	6.003046	0.001258	0.010472	Down
PIK3R4	-1.55331	6.111865	0.000114	0.003328	Down
PRKAR1A	-1.5935	8.269447	0.00126	0.010472	Down
CHMP2B	-1.60272	7.565606	0.004011	0.019562	Down
BNIP3L	-1.6521	7.071348	0.012464	0.039633	Down
VAMP7	-2.10638	6.577966	0.000174	0.003991	Down
NAMPT	-2.25166	7.609224	1.18E-06	0.00072	Down

### Functional enrichment analysis of DEARGs

**Figure 4:**
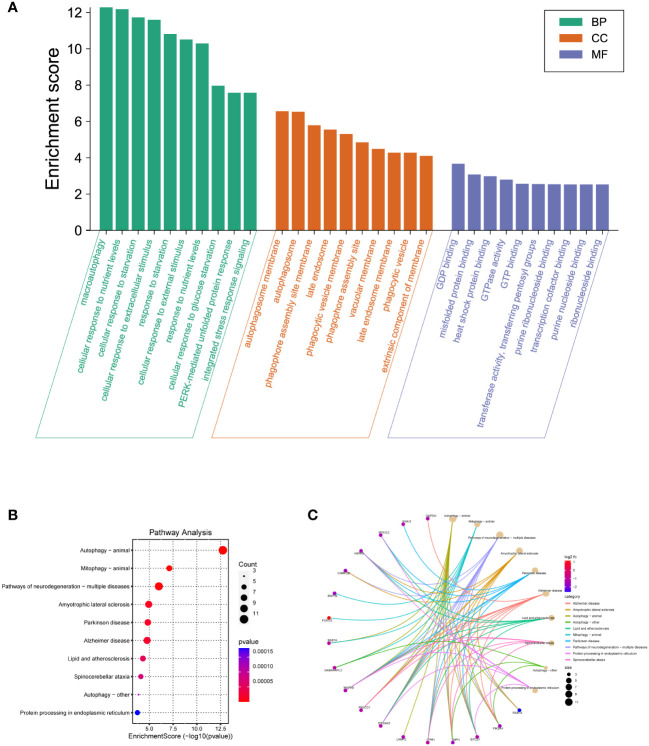
GO and KEGG pathway enrichment analyses of DEARGs. **(A)** The top 10 BP, MF, and CC terms of 30 DEARGs. **(B)** Scatter plots of the top 10 enriched KEGG pathways of 30 DEARGs. **(C)** Plots of KEGG pathway show the “pathway–gene” network.

Since there was a distinct expression pattern between DM2 and normal group, we next investigated the potential biological mechanisms and pathways. The top 10 enriched GO terms of BP, CC, and MF are demonstrated in **Figure 4A** and **Supplementary Table S2**. For BP, the most DEARGs were enriched in macroautophagy, cellular response to nutrient levels, and cellular response to starvation. For CC, the DEARGs were mainly enriched in autophagosome membrane and autophagosome and phagophore assembly site membrane. For MF, the most DEARGs were enriched in GDP binding, misfolded protein binding, and heat shock protein binding. For KEGG pathway analysis, autophagy animal, mitophagy animal, and pathways of neurodegeneration-multiple diseases were the most enriched pathways of DEARGs (**Figure 4B**, **Supplementary Table S3**). Additionally, the “pathway–gene interaction network” was constructed to give a readable visual representation of the complex relationship between DEARGs and KEGG pathways (**Figure 4C**).

### PPI network construction and identification of hub DEARGs

**Figure 5:**
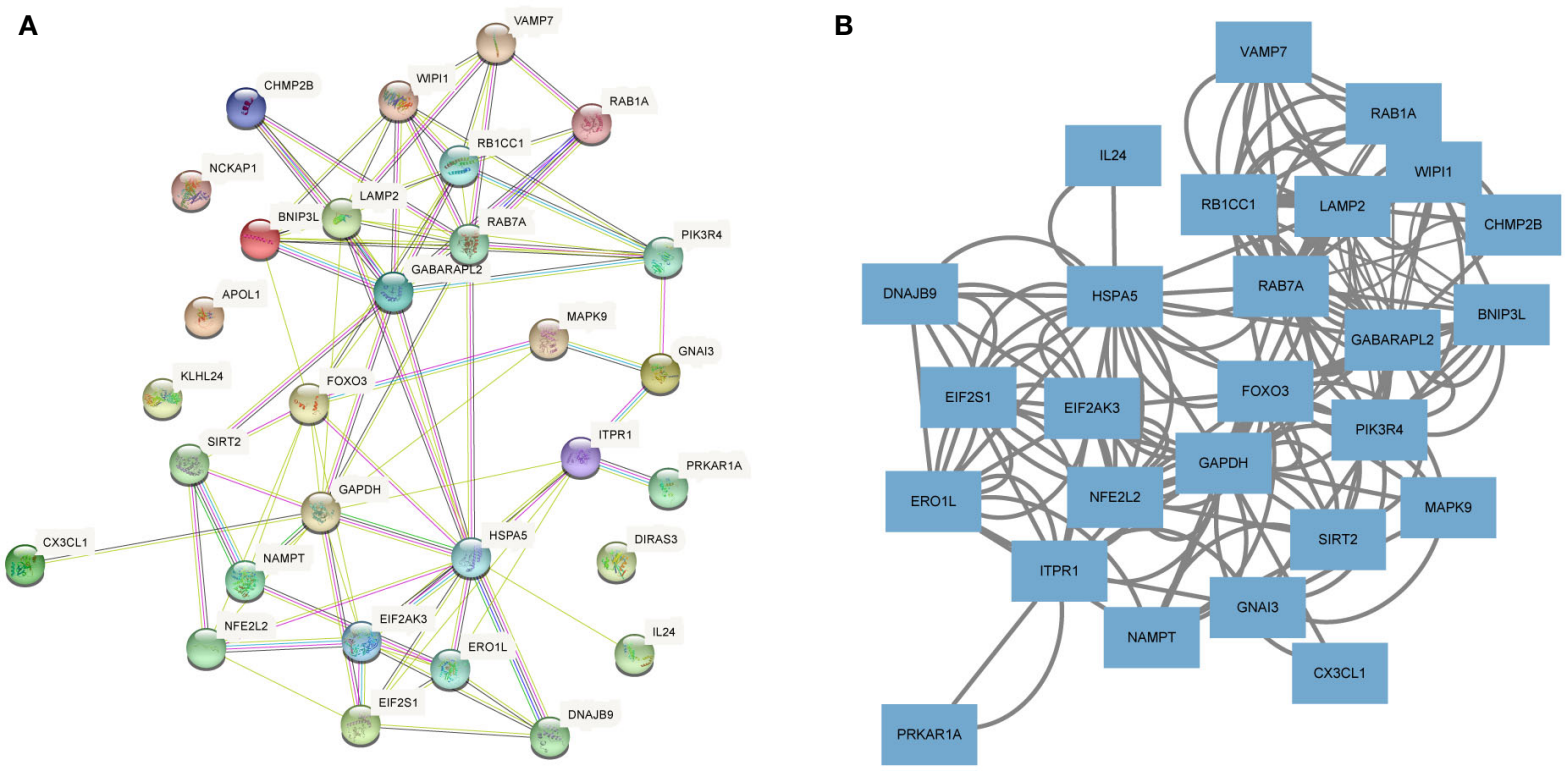
Protein–protein interaction (PPI) analyses of DEARGs. **(A)** The PPI network of 30 DEARGs is analyzed using STRING database. **(B)** The PPI network of 30 DEARGs is analyzed using Cytoscape.

**Figure 6:**
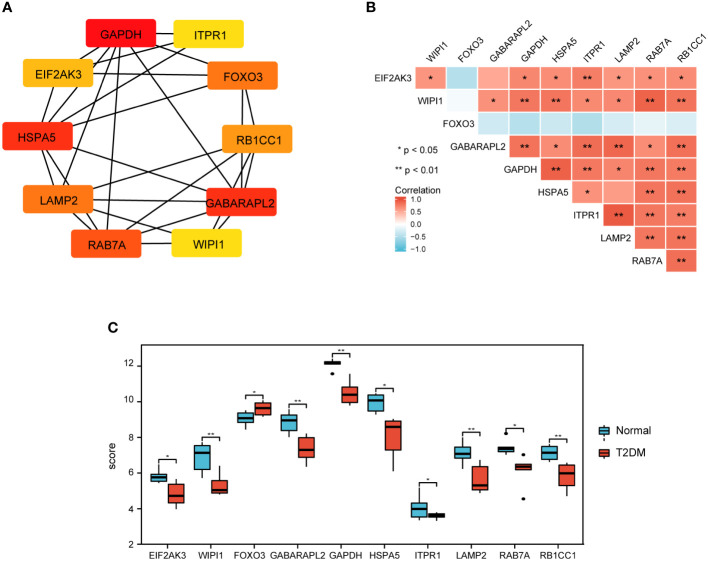
Top 10 key DEARGs identification and expression. **(A)** The network of top 10 DEARGs. **(B)** The expression of top 10 DEARGs in normal and T2DM islet samples *via* bioinformation analysis. **(C)** Correlation among the top 10 DEARGs by Spearman correlation analysis.

To investigate the interactions between the 30 DEARGs, STRING database was used to establish the PPI pairs. Next, MCODE algorithm was employed to identify a sub-network (as shown in **Figure 5A**), containing 30 nodes and 83 edges. Cytoscape was used to visualize the PPI network (**Figure 5B**). The interaction network between the top 10 hub DEARGs was constructed by CytoHubba plugin based on their degree, including GAPDH, ITPR1, EIF2AK3, FOXO3, HSPA5, RB1CC1, LAMP2, GABARAPL2, RAB7A, and WIPI1 (**Figure 6A**). The connectivity degree of top 10 hub DEARGs is detailed in **Table 3**. Then, the correlation analysis of these DEARGs was conducted. There were positive correlations between the expressions of these genes (**Figure 6B**). Moreover, expressions of these 10 DEARGs were evaluated in normal and T2DM samples. We found that the levels of EIF2AK3, WIPI1, GABARAPL2, GAPDH, HSPA5, ITPR1, LAMP2, RAB7A, and RB1CC1 were decreased, while the expression of FOXO3 was increased in T2DM samples (**Figure 6C**).

**Table 3:** Connectivity degree values of top 10 hub DEARGs within the PPI network.

Gene symbol	Gene description	Degree
GAPDH	Glyceraldehyde-3-phosphate dehydrogenase	26
HSPA5	Heat shock protein family a member 5	24
GABARAPL2	GABA type A receptor associated protein like 2	24
RAB7A	Member RAS oncogene family	22
FOXO3	Forkhead box O3	20
LAMP2	Lysosomal-associated membrane protein 2	20
RB1CC1	RB1-inducible coiled-coil 1	18
EIF2AK3	Eukaryotic translation initiation factor 2 alpha kinase 3	16
ITPR1	Inositol 1,4,5-trisphosphate receptor type 1	14
EIF2S1	Eukaryotic translation initiation factor 2 subunit alpha	14

### Expression of hub DEARGs

**Figure 7:**
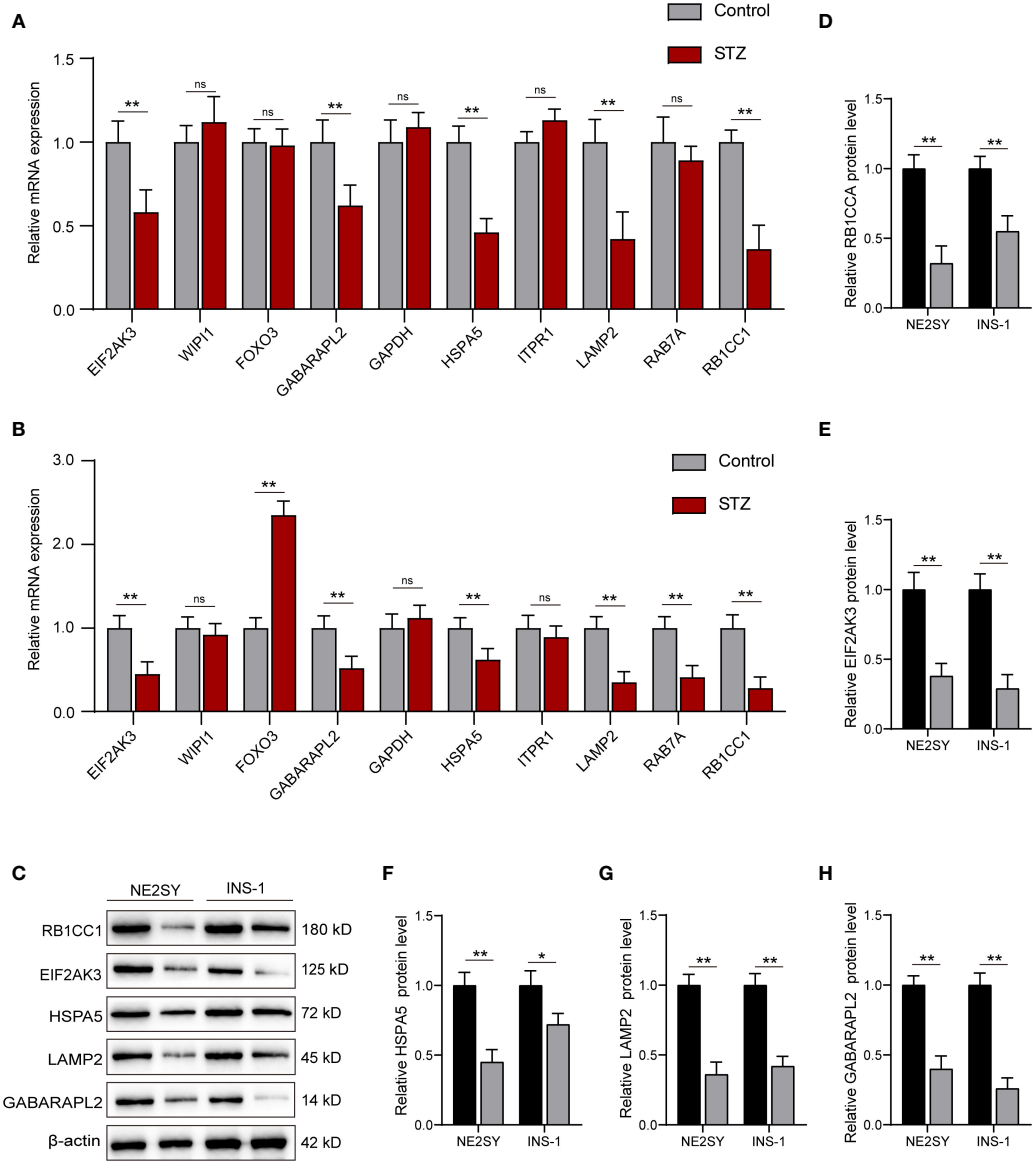
RT-qPCR and Western blot validation of the DEARGs in STZ-treated islet cells. **(A)** Relative mRNA expression of top 10 DEARGs in STZ-treated human islet cells and controls. **(B)** Relative mRNA expression of top 10 DEARGs in STZ-treated rat islet cells and controls. **(C)** The protein expression level of EIF2AK3, GABARAPL2, HSPA5, LAMP2, and RB1CC1 after treatment with STZ in NE2SY and INS-1 cells. **(D)** Protein expression statistics of RB1CC1. **(E)** Protein expression statistics of EIF2AK3. **(F)** Protein expression statistics of HSPA5. **(G)** Protein expression statistics of LAMP2. **(H)** Protein expression statistics of GABARAPL2. ns, not significant; *p<0.05, **p<0.01.

Next, we investigated the expression of top 10 hub DEARGs by using qRT-PCR in NES2Y and INS-1 cells with or without STZ treatment. Among these 10 hub DEARGs, EIF2AK3, GABARAPL2, HSPA5, LAMP2, and RB1CC1 were significantly downregulated compared to the control group in the NE2SY cells (**Figure 7A**). The FOXO3 gene was upregulated, while the levels of EIF2AK3, GABARAPL2, HSPA5, LAMP2, RAB7A, and RB1CC1 genes were downregulated in INS-1 cells under STZ (**Figure 7B**). Accordingly, we focused on five autophagy-related genes both differentially expressed in the two cell types, including EIF2AK3, GABARAPL2, HSPA5, LAMP2, and RB1CC1. We then assessed the effect of STZ on expression these proteins. It was observed that STZ treatment significantly reduced the protein levels of EIF2AK3, GABARAPL2, HSPA5, LAMP2, and RB1CC1 in both NE2SY and INS-1 cells (**Figures 7C–H**).

### Effect of overexpression of EIF2AK3 and RB1CC1 on ARGs

**Figure 8:**
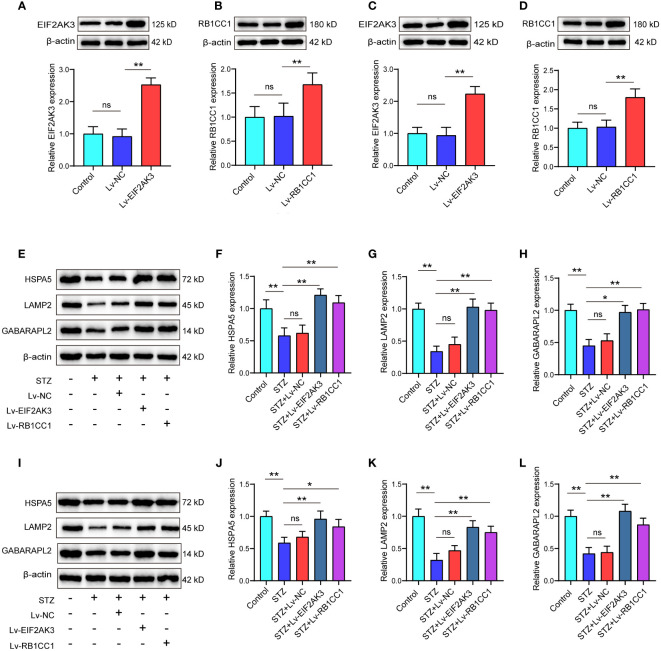
Effect of overexpression of EIF2AK3 and RB1CC1 on ARGs. **(A, B)** Validation of EIF2AK3 or RB1CC1 overexpression in NES2Y cells using Western blot analysis. **(C, D)** Validation of EIF2AK3 or RB1CC1 overexpression in INS-1 cells using Western blot analysis. **(E–H)** Protein levels of GABARAPL2, HSPA5, and LAMP2 after overexpression of EIF2AK3 or RB1CC1 in STZ-treated NES2Y cells. **(I–L)** Protein levels of GABARAPL2, HSPA5, and LAMP2 after overexpression of EIF2AK3 or RB1CC1 in STZ-treated INS-1 cells. ns, not significant; *p<0.05, **p<0.01.

To explore the effects of DEARGs on islet cells, we constructed the EIF2AK3 or RB1CC1 overexpression lentiviral plasmid. After cell transfection, the EIF2AK3 or RB1CC1 was significantly elevated by the transfection with overexpression plasmid in NE2SY cells (**Figures 8A, B**) and in INS-1 cells (**Figures 8C, D**). We further determined the effects of EIF2AK3 or RB1CC1 overexpression on other DEARGs, including GABARAPL2, HSPA5, and LAMP2. Western blot analysis revealed that overexpression of EIF2AK3 or RB1CC1 induced the increased expression of GABARAPL2, HSPA5, and LAMP2 in NE2SY cells (**Figures 8E–H**). Similar trends were observed in INS-1 cells (**Figures 8I–L**).

### Effect of overexpression of EIF2AK3 and RB1CC1 on cell viability and insulin secretion

**Figure 9:**
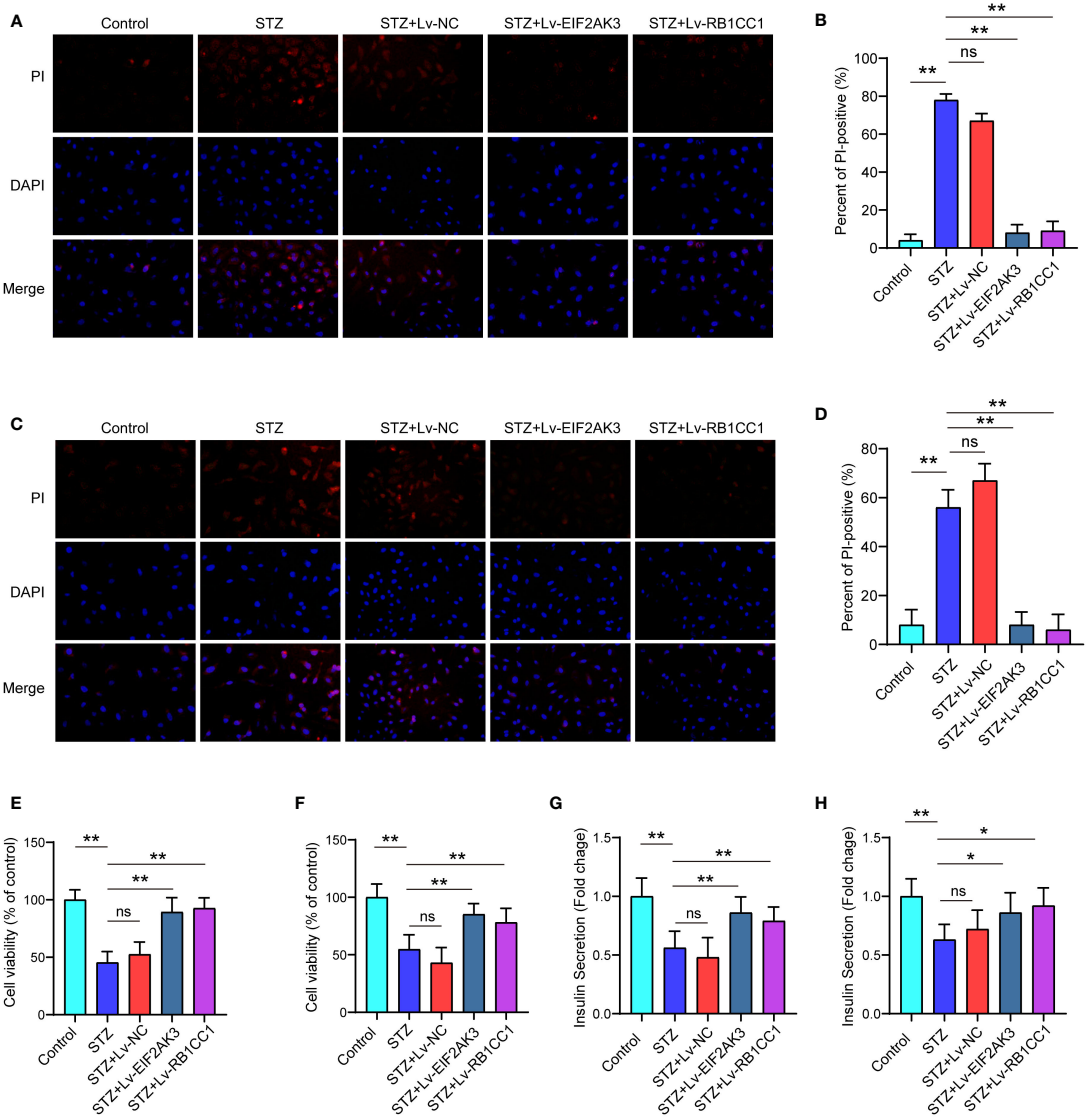
Effect of overexpression of EIF2AK3 and RB1CC1 on cell viability and insulin secretion. **(A)** Representative photomicrographs of PI staining in NES2Y cells. **(B)** Quantification of PI staining in NES2Y cells. **(C)** Representative photomicrographs of PI staining in INS-1 cells. **(D)** Quantification of PI staining in INS-1 cells. **(E, F)** Alterations of cell viability of STZ-treated NES2Y and INS-1 cells after overexpression of EIF2AK3 or RB1CC1. **(G, H)** Changes in the secretion of insulin in STZ-treated NES2Y and INS-1 cells after overexpression of EIF2AK3 or RB1CC1. ns, not significant; *p<0.05, **p<0.01.

We further assessed the effects of EIF2AK3 or RB1CC1 overexpression on cell viability. As shown in **Figures 9A–D**, PI staining showed that STZ treatment significantly increased the amount of PI-positive cells of NES2Y cells or INS-1 cell compared with the control group. Both of EIF2AK3 or RB1CC1 overexpression significantly reduced the percentage of PI-positive cells. The cck8 experiments and PI staining shared a consistent result (**Figures 9E, F**). Subsequently, insulin secretion was measured after overexpression of EIF2AK3 and RB1CC1. Administration of STZ inhibited insulin secretion in NES2Y or INS-1 cells, while overexpression of EIF2AK3 or RB1CC1 markedly increased insulin secretion (**Figures 9G, H**).

## Discussion

Diabetes is a typically chronic disease and is often accompanied with various severe complications, including diabetic nephropathy/vasculopathy/neuropathy and diabetic foot ulcers (22). Lifestyle modification (such as diet and exercise) and oral antidiabetic drugs (such as metformin) are common intervention in the treatment of T2DM (23). These treatments can only decrease blood glucose concentrations; however, β-cell impairment is irreversible (24). Therefore, it is vital to develop alternative therapeutic approaches and agents for T2DM.

Naguib et al. have found that the decreased serum level of the autophagy biomarker Beclin-1 is associated with diabetic kidney disease (DKD) and the degree of albuminuria, which can be used as an indicator for patients with DKD (25). Other previously published researchers have identified biomarkers that are probably implicated in the regulation of autophagy pathway. For instance, a previous study has screened serum circulating miRNAs as potentially predictive biomarkers of T2DM in prediabetic patients, and their target genes are related to autophagy of muscles (26). Meanwhile, Bai and colleagues have identified circular RNAs that may regulate the autophagy of pancreatic β cells *via* interactions with miRNA in T2DM (27). He et al. have discovered five hub genes that are enriched in autophagy, which may be associated with the T2DM-related Alzheimer’s disease (28). In this study, we used 222 ARGs acquired from the Human Autophagy Database that provides a comprehensive list of autophagy-related genes and contains references to numerous genome and protein databases (29).. Next, 30 DEARGs were identified at the intersection of 1,270 T2DM-related DEGs and ARGs. The 30 DEARGs were discovered to be associated with autophagy- and mitophagy-related pathways. Through the PPI network, the top 10 DEARGs have been identified as hub DEARGs (GAPDH, ITPR1, EIF2AK3, FOXO3, HSPA5, RB1CC1, LAMP2, GABARAPL2, RAB7A, and WIPI1). Further experimental studies have demonstrated the role of representative hub DEARGs in T2DM. Our study directly focused on genes associated with autophagy and revealed their role in T2DM, which shared novel ideas for autophagy-related underlying mechanism in T2DM.

Autophagy is a vital safeguard mechanism under normal blood glucose levels; however, autophagy level was downregulated under high blood glucose (30). During the course of T2DM development, lipotoxicity increased pancreatic β-cell intracellular lipid content, subsequently resulting in decreased expression of ARGs and then disturbing steady-state autophagy or autophagic flow (31). Cavener et al. demonstrated that knocking down of EIF2AK3 in tissue or cells resulted in develop significant diabetes (32). In addition, EIF2AK3 has been recognized as an endoplasmic reticulum-related protein involved in β-cell death (33). These findings suggest that downregulated EIF2AK3 may induce the dysfunction of autophagy and β-cell death, therefore leading to T2DM. Autophagy-related 8 can be divided into LC3 subfamily and GABARAP subfamily, the latter in turn subdivided into GABARAP, GABARAPL1, and GABARAPL2 (34). LC3s participate in phagophore membrane elongation, while the GABARAP/GATE-16 subfamily is critically important for later stages of autophagosome maturation (35). GABARAPL2 has not been reported in T2DM, but there are many reports about GABARAPL2 in neurodegenerative disease. Our study first reported GABARAPL2 as a key ARGs contributing to T2DM. One study reported that miR-181b-5p could inhibit starvation-induced cardiomyocyte autophagy *via* targeting Hspa5 (36). Lysosomal-associated membrane protein 2 (LAMP2) is one of the most abundant parts of lysosomal membrane components, which functions as a marker of lysosome density and integrity (37). We demonstrated that the LAMP2 was suppressed in T2DM by experiment validation, which might indicate that downregulated LAMP2 affected the density and integrity of lysosome during T2DM development. Under metabolic stress, RAB7A inhibits pancreatic β-cell apoptosis and autophagy through mediated growth factor receptor trafficking (38). RB1-inducible coiled-coil 1 (RB1CC1) is a member of the ULK1-ATG13-RB1CC1/FIP200 complex protein and is suppressed by mTOR and is a key autophagy inducer (39). Collectively, these identified hub DEARGs may be associated with T2DM occurrence and development. Autophagy is divided into three categories: macroautophagy, microautophagy, and chaperone-mediated autophagy (40). Further GO enrichment analysis in the present study showed that the DEARGs were highly enriched in macroautophagy and cellular response to starvation. KEGG pathway enrichment analysis showed that DEARGs were associated with autophagy and mitophagy. The results from functional enrichment analysis confirmed the regulatory role of these DEARGs in autophagy.

It is noticed that there is no significant change in the expression of WIPI1 or GAPDH between T2DM cells and control cells. WIPI1 is a member of the WIPI family and can be spliced into two isoforms including WIPI1α and WIPI1β. WIPI1α has been demonstrated to slightly bind with autophagy-related 16 like 1 (ATG16L1) that is a key factor for ATG12–ATG5−ATG16L1 complex, leading to the initiation of nascent autophagosomes, whereas the characteristic of WIPI1β has not been clarified (37 ). Hence, it is speculated that WIPI1 may be spliced into WIPI1β in T2DM cells and control cells, which results in no significant change in the expression of WIPI1 between these two groups. GAPDH is a well-known common enzyme with uncommon functions in the cytoplasm. GAPDH has been noticed to be interacted with considerable partners, such as proteins, various RNA species, and telomeric DNA (41). These interactions may affect the level of GAPDH in T2DM cells and control cells.

Nevertheless, some limitations should be noticed in this study. We retrieved microarray expression profiles of six T2DM islet samples from the GEO database. These retrospective data might induce selection bias due to the small sample size, and therefore, further prospective studies with large sample size should be performed to validate our results. Although we have quantified the expression of hub DEARGs and evaluated the effects of representative DEARGs on islet cells, their underlying regulatory mechanisms in T2DM should be extensively investigated *in vivo* and *in vitro*. Whether WIPI1 is spliced into WIPI1α or WIPI1β needs to be explored in the future. The underlying mechanism of interaction between GAPDH and other partners in T2DM cells should be further investigated.

In conclusion, 30 potential DEARGs were identified and 10 hub DEARGs were discovered to be involved in autophagy- and mitophagy-related pathways. Moreover, the key autophagy-related genes EIF2AK3, GABARAPL2, HSPA5, LAMP2, and RB1CC1 were both differentially expressed in NES2Y and INS-1 cells. Experimental studies deciphered that overexpression of EIF2AK3 or RB1CC1 promoted the cell viability of islet cells and increased the insulin secretion. This study might shed novel insights of DEARGs into T2DM and provide potential biomarkers as therapeutic targets for T2DM.

## Data availability statement

The original contributions presented in the study are included in the article/**Supplementary Material**. Further inquiries can be directed to the corresponding author.

## Author contributions

KC performed data analysis and wrote the manuscript. ZL designed the study and drafted the manuscript. All authors contributed to the article and approved the submitted version.
