# Supplementary material for: Identification and analysis of type 2 diabetes-mellitus-associated autophagy-related genes
Source: Front Endocrinol (Lausanne). 2023 May 8;14:1164112. doi: 10.3389/fendo.2023.1164112 (PMC10200926; doi:10.3389/fendo.2023.1164112)
Supplement: Supplementary file 1 [file Table_1.docx]

Supplementary Material

Identification and analysis of type 2 diabetes mellitus-associated autophagy-related genes

**Kun Cui^1^, Zhizheng Li^2^***

*** Correspondence:** Zhizheng Li: [drli_zhizheng@163.com](mailto:drli_zhizheng@163.com)

**Supplementary Table 1**

Supplementary Table 1: The biological functions of 30 DEARGs

| **DEARGs** | **Gene description** | **Functions** |
| --- | --- | --- |
| KLHL24 | Kelch-like protein 24 | Necessary to maintain the balance between intermediate filament stability and degradation, a process that is essential for skin integrity |
| SIRT2 | NAD-dependent protein deacetylase sirtuin-2 | Participates in the modulation of multiple and diverse biological processes such as cell cycle control, genomic integrity, microtubule dynamics, cell differentiation, metabolic networks, and autophagy |
| CX3CL1 | C-X3-C motif chemokine ligand 1 | Chemokine that acts as a ligand for CX3CR1 |
| APOL1 | Apolipoprotein L1 | Lipid exchange and transport |
| FOXO3 | Forkhead Box O3 | Acts as a positive regulator of autophagy |
| ITPR1 | Inositol 1,4,5-Trisphosphate Receptor Type 1 | Mediates calcium release from the endoplasmic reticulum |
| RAB7A | Member RAS Oncogene Family | Regulation of endo-lysosomal trafficking |
| ERO1L | ERO1-like protein alpha | Oxidoreductase involved in disulfide bond formation in the endoplasmic reticulum |
| GABARAPL2 | GABA Type A Receptor Associated Protein Like 2 | Ubiquitin-like modifier involved in intra-Golgi traffic |
| EIF2AK3 | Eukaryotic Translation Initiation Factor 2 Alpha Kinase 3 | Control of mitochondrial morphology and function |
| NCKAP1 | Nck-associated protein 1 | Regulates lamellipodia formation |
| RB1CC1 | RB1 Inducible Coiled-Coil 1 | Regulates autophagosome formation through direct interaction with Atg16L1 |
| GNAI3 | Guanine nucleotide-binding protein G(k) subunit alpha | As transducers downstream of G protein-coupled receptors in numerous signaling cascades. |
| GAPDH | Glyceraldehyde-3-Phosphate Dehydrogenase | Catalyzes the first step of the pathway by converting G3P into 3-phospho-D-glyceroyl phosphate |
| EIF2S1 | Eukaryotic Translation Initiation Factor 2 Subunit Alpha | Functions in the early steps of protein synthesis by forming a ternary complex with GTP and initiator tRNA |
| MAPK9 |  | Dual role in suppressing or encouraging autophagy |
| IL24 | Interleukin-24 | Has antiproliferative properties on melanoma cells and may contribute to terminal cell differentiation |
| DIRAS3 | DIRAS Family GTPase 3 | Induce autophagy in human by blocking PI3K signaling, inhibiting the mammalian target of rapamycin (TOR), upregulating ATG4, and colocalizing with LC3 in autophagosomes |
| NFE2L2 | NFE2 Like BZIP Transcription Factor 2 | Important for the coordinated up-regulation of genes in response to oxidative stress |
| LAMP2 | Lysosomal Associated Membrane Protein 2 | Mediates lysosomal degradation |
| HSPA5 | Heat Shock Protein Family A Member 5 | Plays a role in facilitating the assembly of multimeric protein complexes inside the endoplasmic reticulum |
| DNAJB9 | DnaJ homolog subfamily B member 9 | Acts as a co-chaperone with an Hsp70 protein |
| WIPI1 | WD repeat domain phosphoinositide-interacting protein 1 | Plays an important role in autophagy and in particular starvation- and calcium-mediated autophagy, as well as in mitophagy. |
| PIK3R4 | Phosphoinositide 3-kinase regulatory subunit 4 | Involved in initiation of autophagosomes and maturation of autophagosomes and endocytosis. |
| PRKAR1A | cAMP-dependent protein kinase type I-alpha regulatory subunit | Regulatory subunit of the cAMP-dependent protein kinases involved in cAMP signaling in cells |
| CHMP2B | Charged multivesicular body protein 2b | core component of the endosomal sorting required for transport complex III (ESCRT-III) which is involved in multivesicular bodies (MVBs) formation and sorting of endosomal cargo proteins into MVBs |
| BNIP3L | BCL2/adenovirus E1B 19 kDa protein-interacting protein 3-like | Interacts with cellular anti-apoptosis proteins |
| VAMP7 | Vesicle-associated membrane protein 7 | Involved in the targeting and/or fusion of transport vesicles to their target membrane during transport of proteins from the early endosome to the lysosome |
| NAMPT | Nicotinamide phosphoribosyltransferase | Catalyzes the condensation of nicotinamide with 5- phosphoribosyl-1-pyrophosphate to yield nicotinamide mononucleotide, an intermediate in the biosynthesis of NAD |

**Supplementary Table 2**

Supplementary Table 2: A list of the top10 enriched GO terms

| **ID** | **Description** | **GeneRatio** | **BgRatio** | **pvalue** | **p.adjust** |
| --- | --- | --- | --- | --- | --- |
| GO:0016236 | macroautophagy | 11/29 | 310/18866 | 5.2536E-13 | 4.7927E-10 |
| GO:0031669 | cellular response to nutrient levels | 10/29 | 221/18866 | 6.54294E-13 | 4.7927E-10 |
| GO:0009267 | cellular response to starvation | 9/29 | 163/18866 | 1.85507E-12 | 9.0589E-10 |
| GO:0031668 | cellular response to extracellular stimulus | 10/29 | 253/18866 | 2.52171E-12 | 9.23576E-10 |
| GO:0042594 | response to starvation | 9/29 | 206/18866 | 1.53528E-11 | 4.49838E-09 |
| GO:0071496 | cellular response to external stimulus | 10/29 | 326/18866 | 3.09719E-11 | 7.56232E-09 |
| GO:0031667 | response to nutrient levels | 11/29 | 473/18866 | 5.04679E-11 | 1.05622E-08 |
| GO:0042149 | cellular response to glucose starvation | 5/29 | 49/18866 | 1.0857E-08 | 1.98818E-06 |
| GO:0036499 | PERK-mediated unfolded protein response | 4/29 | 21/18866 | 2.64572E-08 | 3.87598E-06 |
| GO:0140467 | integrated stress response signaling | 4/29 | 21/18866 | 2.64572E-08 | 3.87598E-06 |
| GO:0000421 | autophagosome membrane | 4/29 | 38/19559 | 2.77763E-07 | 2.46866E-05 |
| GO:0005776 | autophagosome | 5/29 | 97/19559 | 2.92149E-07 | 2.46866E-05 |
| GO:0034045 | phagophore assembly site membrane | 3/29 | 16/19559 | 1.61995E-06 | 9.12575E-05 |
| GO:0005770 | late endosome | 6/29 | 278/19559 | 2.81907E-06 | 0.000119106 |
| GO:0030670 | phagocytic vesicle membrane | 4/29 | 77/19559 | 4.89311E-06 | 0.000165387 |
| GO:0000407 | phagophore assembly site | 3/29 | 32/19559 | 1.41211E-05 | 0.000397744 |
| GO:0005774 | vacuolar membrane | 6/29 | 427/19559 | 3.2425E-05 | 0.000782832 |
| GO:0031902 | late endosome membrane | 4/29 | 140/19559 | 5.19679E-05 | 0.000975841 |
| GO:0045335 | phagocytic vesicle | 4/29 | 140/19559 | 5.19679E-05 | 0.000975841 |
| GO:0019898 | extrinsic component of membrane | 5/29 | 306/19559 | 7.91477E-05 | 0.001337596 |
| GO:0019003 | GDP binding | 3/29 | 74/18352 | 0.000213266 | 0.030923597 |
| GO:0051787 | misfolded protein binding | 2/29 | 27/18352 | 0.0008258 | 0.035207745 |
| GO:0031072 | heat shock protein binding | 3/29 | 127/18352 | 0.001036843 | 0.035207745 |
| GO:0003924 | GTPase activity | 4/29 | 326/18352 | 0.001634818 | 0.035207745 |
| GO:0005525 | GTP binding | 4/29 | 376/18352 | 0.002746398 | 0.035207745 |
| GO:0016763 | transferase activity, transferring pentosyl groups | 2/29 | 50/18352 | 0.00281793 | 0.035207745 |
| GO:0032550 | purine ribonucleoside binding | 4/29 | 380/18352 | 0.002853171 | 0.035207745 |
| GO:0001221 | transcription cofactor binding | 2/29 | 51/18352 | 0.002930082 | 0.035207745 |
| GO:0001883 | purine nucleoside binding | 4/29 | 383/18352 | 0.002935103 | 0.035207745 |
| GO:0032549 | ribonucleoside binding | 4/29 | 383/18352 | 0.002935103 | 0.035207745 |

**Supplementary Table 3**

Supplementary Table 3: A list of top 10 KEGG pathways

| **ID** | **Description** | **GeneRatio** | **BgRatio** | **pvalue** | **p.adjust** |
| --- | --- | --- | --- | --- | --- |
| hsa04140 | Autophagy - animal | 11/26 | 141/8115 | 1.81912E-13 | 2.63772E-11 |
| hsa04137 | Mitophagy - animal | 6/26 | 72/8115 | 7.89897E-08 | 5.72675E-06 |
| hsa05022 | Pathways of neurodegeneration - multiple diseases | 10/26 | 476/8115 | 9.94286E-07 | 4.80571E-05 |
| hsa05014 | Amyotrophic lateral sclerosis | 8/26 | 364/8115 | 1.16411E-05 | 0.000416017 |
| hsa05012 | Parkinson disease | 7/26 | 266/8115 | 1.4476E-05 | 0.000416017 |
| hsa05010 | Alzheimer disease | 8/26 | 384/8115 | 1.72145E-05 | 0.000416017 |
| hsa05417 | Lipid and atherosclerosis | 6/26 | 215/8115 | 4.76394E-05 | 0.000986817 |
| hsa05017 | Spinocerebellar ataxia | 5/26 | 143/8115 | 7.73277E-05 | 0.001401565 |
| hsa04136 | Autophagy - other | 3/26 | 32/8115 | 0.000136178 | 0.002193982 |
| hsa04141 | Protein processing in endoplasmic reticulum | 5/26 | 171/8115 | 0.000180001 | 0.00261002 |
